# Dataset on the innovation remediation technology of embankment dams by using suitable types of alternative raw materials

**DOI:** 10.1016/j.dib.2018.01.092

**Published:** 2018-02-05

**Authors:** Drochytka Rostislav, Kociánová Magdaléna

**Affiliations:** Institumaterials” te of Technology of Building Materials and Components, Faculty of Civil Engineering, Brno University of Technology, Veveří 95, 602 00 Brno, Czech Republic

## Abstract

The data presented in this article are related to the research article entitled “Options for the remediation of embankment dams using suitable types of alternative raw (Drochytka and Kociánová, 2017) [1]. This article describes the possibility of use the fly ash as an optimal alternative material that can reduces costs and improves the rheological properties of the grouts. The grout data set is made publicly available to enable critical or extended analyses.

**Specifications Table**

**Table:** 

Subject area	*Civil engineering*
More specific subject area	*Effect of fly ash on grout to remediation embankment dams*
Type of data	*Figures, Text file*
How data was acquired	*Conducting of research in designing the optimal grout for remediation of embankment dams in global scale. The viscosity was observed with Execution of special geotechnical work – Grouting method. Compressive strength and volumetric shrinkage were measured based on standards. The influence of fly ash on behaving the grouts in raw (viscosity) and hardened state (compressive strength, volumetric shrinkage) were measured.*
Data format	*Analysed, measured*
Experimental factors	*GE (green earth) clay together with two types of ash (FA – fly ash, FBC - fluidised bed combustion ash) and lime (L) were selected for the design of mixtures. The effect of fly ash on the designed mixtures to reduce the leaks in the dam was determined.*
Experimental features	*The relationships between the GE clay, two types of fly ash and lime for to design the optimal remediation grout were determined.*
Data source location	*Brno, Czech Republic, The Cheb basin*
Data accessibility	*The data are available within this article*

**Value of the data**

• The data presents the optimized technology of remediation embankment dams by suitable alternative raw materials and could be used by others researchers.
• The rheological properties of grouts were measured by using Mash cone and were compared to others chemical grouts.
• This data allows other researchers to extend the statistical analyses.

## 1 Data

**Fig. 1:**
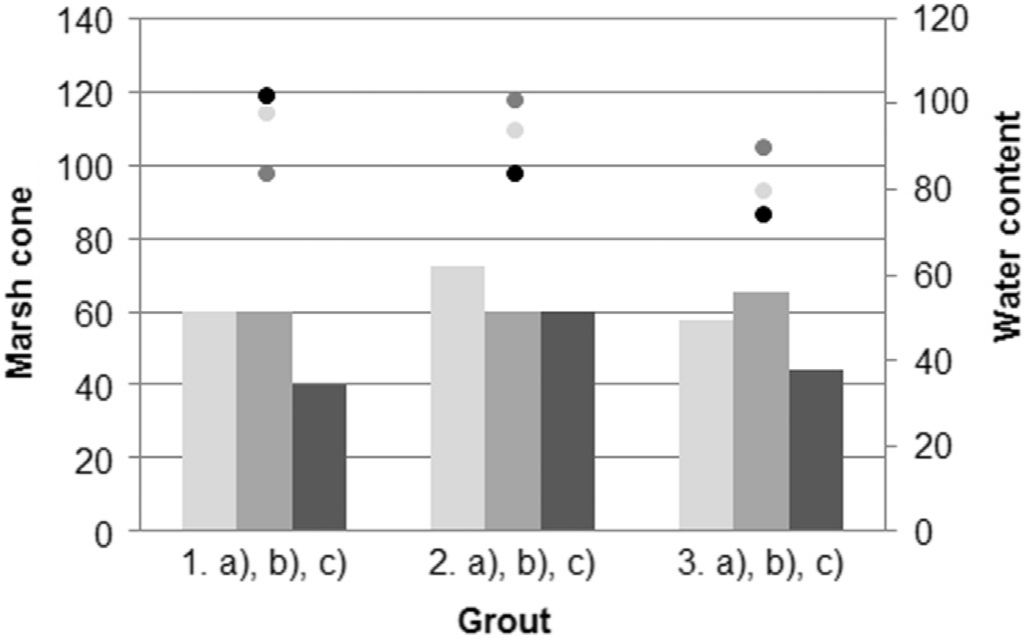
Dependence of viscosity on the water content of mixtures based on GE clay, FA, FBC and lime (1.a) GE, 2.a) GE + 25 FA, 3.a) GE + 50 FA, 1.b) GE, 2.b) GE + 25 FBC, 3.b) GE + 50 FBC, 1.c) GE + L, 2.c) GE + 25 FA + L, 3. c) GE + 50 FA + L).

**Fig. 2:**
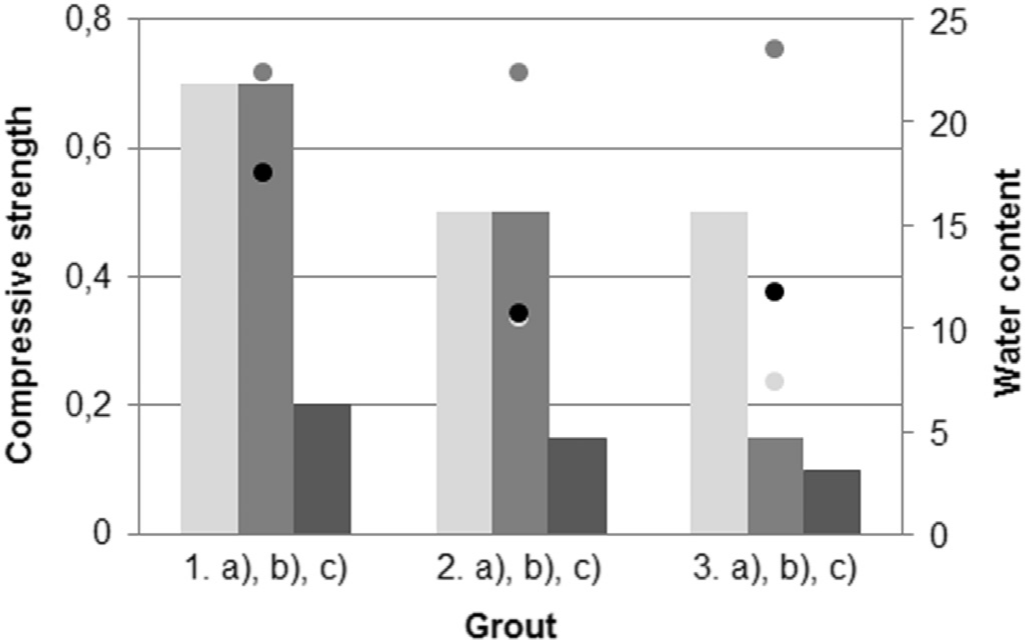
The determination of the compressive strength based on GE clay, fly ash and lime (1.a) GE, 2.a) GE + 25 FA, 3.a) GE + 50 FA, 1.b) GE, 2.b) GE + 25 FBC, 3.b) GE + 50 FBC, 1.c) GE + L, 2.c) GE + 25 FA + L, 3. c) GE + 50 FA + L).

**Fig. 3:**
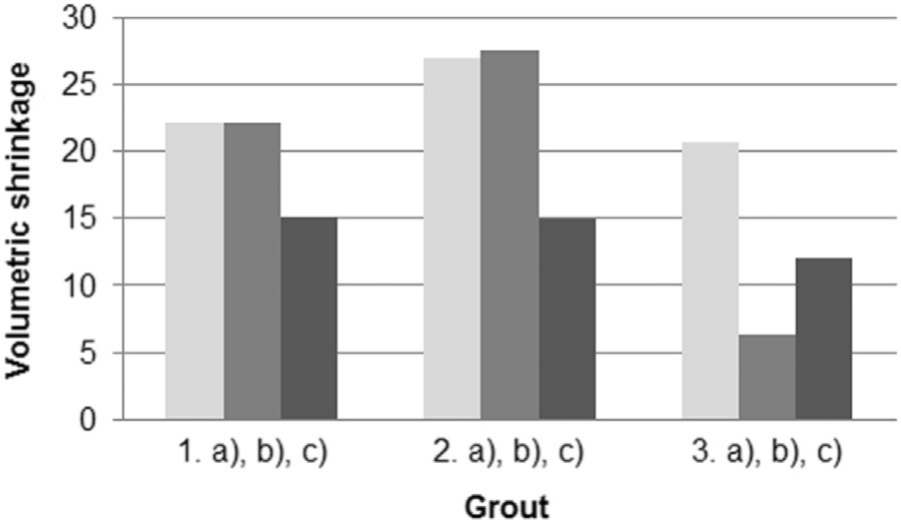
The results of the determination of the volumetric shrinkage of samples after 28 days of hardening (1.a) GE, 2.a) GE + 25 FA, 3.a) GE + 50 FA, 1.b) GE, 2.b) GE + 25 FBC, 3.b) GE + 50 FBC, 1.c) GE + L, 2.c) GE + 25 FA + L, 3. c) GE + 50 FA + L).

The dataset of this article provides information about the innovation remediation technology of embankment dams by use the suitable types of alternative raw materials. [1] Fig. 1 shows the dependence of viscosity on the water content of suspensions based on GE clay (green earth clay), FA (fly ash), FBC (fluidised bed combustion ash) and lime (L). In Fig. 2 are shown the compressive strengths of the samples. On Fig. 3 we can see the determination of the volumetric shrinkage (too contraction) of samples.

## 2 Experimental design, materials & methods

### 2.1 Influence of alternative raw materials on designed remediation grouts measurements

The experiments of use the optimal grout were carried out on several types of embankment dams in the Czech Republic. The experiments carried out even before the winter, due to the fact that the dam was ready for the upcoming winter season and especially for the spring floods caused by melting snow, regional or local rainfall. On designed grouts were monitored the effects of mixtures on leaks of the dams. GE clay together with two types of ash (FA, FBC) and lime (L) were selected for the design of optimal grouts. When designing, the mixtures were combined with the selected types of additives and their quantities. Before the application of grouts in practice, the tests on grouts were performed in raw and hardened state. On designed grouts were measured the viscosity (in raw state), compressive strength and volumetric shrinkage (in hardened state). The viscosity was determined according to EN 12 715 [2]. For determination the test used a Marsh viscometer, which has the shape of a cone. Compressive strength was determined according to CEN ISO/TS 17892-7 [3], [4]. The compressive strength and volumetric shrinkage were tested on cubes 100×100×100 mm, after 28 days. At the end we evaluated the properties of the designed optimal grout and their effect in damaged dams in comparison with traditional grout currently used in practice.
