# Supplementary material for: Dataset on the innovation remediation technology of embankment dams by using suitable types of alternative raw materials
Source: Data Brief. 2018 Feb 5;17:753–6. doi: 10.1016/j.dib.2018.01.092 (PMC5988509; doi:10.1016/j.dib.2018.01.092)
Supplement: Supplementary file 1 — Supplementary material [file mmc1.docx]

Cover Letter

**What is the novelty of this work?**

The novelty of this work is new technology of injection embankment dams. Nowadays clay-cement mixture is usually chosen for remediation of embankment dams. Suitable material composition of grout is important for avoiding or reduction leakage of dams. Nowadays clay-cement mixtures are used because they are better and have a better tendency to penetrate into incoherent sediments. This mixture is expensive and usually does not lead to desire effect. Because, during the injection, a large amount of the mixes is consumed, we must think to cost. To reduce costs the use of power industry by-products and secondary raw materials is required. Above all, it is fly ash, which we used in our experiment. It has been found, that the use of fly ash leads to improvement of consistency of the fresh mixture, reducing the water-cement ratio and shrinkage of the mixture.

**Is the paper appealing to a popular or scientific audience?**

This paper is appealing to a scientific audience.

**Why the authors think the paper is important and why the journal should publish it?**

There are lots of water works in all Europe, where we can use this technology for remediation of embankment dams. After meeting all requirements which are set for the mixture can produce a compact mixture which prevents further leakages of embankment dams.

**Has the article been checked by a native tongue speaker with expertise in the field?**

Yes the article was check by a native tongue speaker with expertise in the field.

**Are you available as a reviewer for at least three other articles for WM during the current year?**

Yea, I am available as a reviewer for at least three other articles for WM during the current year.
